# Does Motor Training of the Nonparetic Side Influences Balance and Function in Chronic Stroke? A Pilot RCT

**DOI:** 10.1155/2014/769726

**Published:** 2014-11-17

**Authors:** Shanta Pandian, Kamal Narayan Arya, Dharmendra Kumar

**Affiliations:** Pandit Deendayal Upadhayaya Institute for the Physically Handicapped, 4 VD Marg, New Delhi 110002, India

## Abstract

*Background*. Balance and functional abilities are controlled by both sides of the body. The role of nonparetic side has never been explored for such skills. *Objective*. The objective of the present study was to examine the effect of a motor therapy program primarily involving the nonparetic side on balance and function in chronic stroke. *Method*. A randomized controlled, double blinded trial was conducted on 39 poststroke hemiparetic subjects (21, men; mean age, 42 years; mean poststroke duration, 13 months). They were randomly divided into the experimental group (*n* = 20) and control group (*n* = 19). The participants received either motor therapy focusing on the nonparetic side along with the conventional program or conventional program alone for 8 weeks (3 session/week, 60 minutes each). The balance ability was assessed using Berg Balance Scale (BBS) and Functional Reach Test (FRT) while the functional performance was measured by Barthel Index (BI). *Result*. After intervention, the experimental group exhibited significant (*P* < 0.05) change on BBS (5.65 versus 2.52) and BI (12.75 versus 2.16) scores in comparison to the control group. *Conclusion*. The motor therapy program incorporating the nonparetic side along with the affected side was found to be effective in enhancing balance and function in stroke.

## 1. Introduction

Stroke demonstrates a group of symptoms including the paresis of the body-side, contralateral to the lesion. Motor weakness on the ipsilesional side is now evident to be an additional consequence [1]. The weakness may exhibit in form of impairment in dexterity, coordination, and muscle strength. The word “less-affected” is now being used interchangeably in clinical practice. The impairment of the nonparetic side is mild and unnoticeable. Hence, the assessment and therapy has never been focused on the side. However, the asymmetry out of the weakness may lead to balance dysfunction during the functional performance. Functional level of stroke subjects had been explored considering the affected extremity and trunk but not regarding the weakness of nonparetic extremities [2–4].

Balance, a multifactorial phenomenon, is an ability to maintain upright and weight bearing posture within the base of support not leading to a fall. The ability is considered to be responsible for various bodily movements during functional performance [5]. For instance, dynamic balance is a prerequisite for various self-care activities [6]. The perturbed body stability and compromised proprioceptive inputs are some of the vital causes for balance impairment among stroke survivors [7, 8]. The balance dysfunction during daily tasks leads to fall, one of the common complications of the stroke. The dysfunction leads to poor quality of life and impedes the functional recovery [9, 10].

During normal human life, balance and functional tasks are fulfilled by both sides of the body in a mutually coordinated manner. After the onset of stroke, the contralesional side is more affected than the ipsilesional side. The contralesional side of body is usually considered to be responsible for poor balance [11]. The trunk also affects balance and related tasks in stroke subjects [4, 12]. The stroke subjects bear more weight on the nonparetic side to compensate the affected side in upright posture [13].

After stroke, majority of the daily activities are expected to be carried out by the ipsilesional side. Now, it is evident that some form of motor deficits persists on the ipsilesional body side [1]. Through majority of the fibers, a cerebral hemisphere controls voluntary movements of the opposite side of the body. However, few proportions of the fibers also have innervations for the same-side movements. This could be the reason for the deficits of nonparetic side in stroke [14–16]. The ability to manipulate objects swiftly by the nonparetic upper limb gets impaired. Further, most of the nonparetic muscles have fair to moderate strength. The deficits may contribute to poor balance and function. The deficits of nonparetic side may lead to balance dysfunction and impairment in performing both bilateral and unilateral functional tasks. Additionally, due to the relation between balance and functional tasks the impairment of nonparetic side double folds the challenges [6].

Every functional task can only be fulfilled by a good balance. In addition to the mutual relation, functional performance and balance skills are also associated with the motor ability of both the body sides. The right and left body sides play their respective role for harmonizing balance and function. Both the arms have a crucial involvement for organizing the dynamic balance [17–19].

The complex linkage between the body sides and balance related tasks makes the poststroke rehabilitation a challenge. Various motor therapies are being applied focusing on the affected side. However, none of them could resolve the risk of minimal fall [20]. The task related activities were found to be more beneficial for improving balance than the specific balance-oriented therapy in poststroke subjects [21].

As per our knowledge, no research demonstrates the role of nonparetic side for balance and function. It is well determined that the balance and daily functions are managed by both the sides of body. However, ipsilesional or nonparetic side has never been explored regarding balance and functional issues. Hence there is a need to assess the effect of motor training focusing on the ipsilesional side on balance and functional tasks. The objective of the present study was to investigate the effect of a motor therapy program on the balance ability and functional performance in chronic poststroke patients. The therapy comprised primarily the nonparetic side along with the affected side.

## 2. Methods

### 2.1. Participants

The pilot trial incorporated 39 poststroke hemiparetic individuals who were attending the outdoor occupational therapy unit of a rehabilitation institute. The diagnosis of stroke was made by a neurophysician based on the radiological findings of a CT scan or MRI [22].

The inclusion criteria for the potential participants were ischemic or hemorrhagic stroke, under 60 years of age, >24 weeks poststroke, either right or left side hemiparesis, Brunnstrom recovery stage (BRS) 2 and above [23], and functional ambulation classification level III (independent walking with verbal supervision) and above [24]. The patients with acute medical illness, neuromusculoskeletal complications on the nonparetic side, uncontrolled hypertension (blood pressure 140/90 mm Hg), and severe cognitive and perceptual deficits were excluded from the study.

The ethics committee of the Pandit Deendayal Upadhyaya Institute for the physically handicapped approved the present study. All the participants signed the informed consent before the commencement of the study.

### 2.2. Design

A double blinded, randomized controlled design was used in this study. The subjects, who met the eligibility criteria, were randomly allocated either to the experimental group or control group by using simple randomization method (computer generated random numbers). A research assistant, blinded to the purpose of study, performed the process of randomization and concealed allocation. Sealed opaque envelops were prepared for the process of allocation. Envelops containing the allocated interventions were serially ordered.

The study participants were blinded for the treatment of interest. The experimental and control group sessions were carried out separately to avoid any diffusion of the therapy. The outcome measures were executed by an assessor who was not the part of present study and had adequate experience in applying such measures.

## 3. Interventions

The control group subjects underwent standard motor rehabilitation (based on Brunnstrom's movement therapy) incorporating the affected side only [23]. The protocol comprised reflexive, synergistic, and out-of-synergy movements of the paretic upper limb and lower limb. The main focus of the intervention was to induce voluntary motor control. Various functional activities such as sit-to-stand, stepping, reaching, and side-ward walking and straight-line walking were also provided. In addition to the control intervention, the experimental protocol comprised motor training of both the sides. The program had two components, resistive exercises for the nonparetic side and bimanual activities, 30 minutes each. The resistive exercises were based on the progressive resistive exercise principle of Delorme and Watkins [25]. The strengthening exercises were performed for the weak muscles (grade 4 or less) of the nonparetic upper and lower limbs. The strength for all the muscle groups (shoulder, elbow, forearm, wrist, hip, knee, and ankle) of nonparetic upper and lower limbs was assessed by manual testing method [26, 27]. In addition to the resistive exercises, various bimanual activities such as arm cycling, rowing, and postural transition were also provided. The protocol was carried out for 8 weeks with a frequency of 3/week. The control group subjects underwent similar duration of therapy.

## 4. Outcome Measures

*Berg Balance Scale (BBS)*. The purpose of BBS is to measure balance impairments and fall risks in stroke subjects. Different functional tasks are being utilized to assess the construct. It is a psychometrically sound measure to assess balance impairment in poststroke subjects [28]. It is a 14-item performance-based measure; each item is scored on a 5-point scale (0, poor balance to 4, good balance) with maximum score of 56. Total time requirement to apply the measure is 15 to 20 minutes. The items range from sit-to-stand to standing on one leg. The main focus of the scale is to assess erect posture and alteration during transition of posture [29]. Test-retest reliability for stroke was recorded as ICC = 0.72 to 0.95 [30, 31] and interrater reliability for stroke as ICC = 0.95 [32]. Internal consistency was exhibited as (*α*) 0.98 [33]. Concurrent validity ranged from *r* = 0.90 to 0.95 while the construct validity varied from *r* = 0.82 to 0.94 [32]. Minimal detectable change for BBS was found to be 4.13 to 4.66 in stroke subjects [30, 31]. Fifteen to twenty minutes are required to observe and apply the scale. The scale is interpreted for low fall risk (41–56), medium fall risk (21–40), and high fall risk (0–20) [29].

*Functional Reach Test (FRT)*. The test is a quickly performed tool for various balance impairments including stroke [34]. A measuring tape is mounted on the wall at the height of acromion process of the subject. The subject is made to stand at arm's length with a fixed base of support. Patient is instructed to raise the arm at 90° in forward direction with fist closed and elbow at 0°. Initial reading is taken at the level of 3rd metacarpal, followed by the leaning in forward direction maintaining the base of support or moving ahead. Second reading is taken using the same reference point and subtracted from the initial reading. In the present study, the assessment was performed on the nonparetic side as the testing procedures were not possible for moderately paretic side (inclusion criteria: BRS 2 and above). Further, this test being a ratio scale was used as a secondary assessment tool for balance in addition to BBS. The tool exhibits excellent reliability (ICC = 0.92 to 0.98) and validity (ICC = 0.65) [35].

*Barthel Index (BI)*. It is an evident and most commonly used measure to quantify the functional changes in stroke subjects [36]. This 10-item performance-based measure assesses the activities of daily living. The scale exclusively measures the degree of assistance required by the subject. Levels of measurement are recorded from complete independence to needing assistance. Each item is assigned a score of 0, 5, 10, or 15; each item is assessed differently [29]. A global score range from 0 to 100 is calculated. The scale demonstrated strong interrater reliability (*r* = 0.95), test-retest reliability (*r* = 0.89), and concurrent validity (*r* = 0.74 to 0.80) [35, 37]. For stroke subjects, the criterion validity was recorded between *r* = 0.83 and 0.92 [38]. The construct validity for stroke is *r* = 0.83 [38–40]. The minimum detectable change and minimal clinical important difference were documented to be 4.02 points and 1.85 points, respectively, in chronic stroke [41].

## 5. Data Analysis

A repeated-measures 2-way ANOVA (continuous data; within factor, time of assessment; between factor, intervention group) was executed to record the difference between the groups for the balance and function measures. The demographic and baseline continuous observations were analyzed using independent *t*-test. However, for nominal/ordinal data chi-square/Fischer exact test was used. All the statistical analysis was carried out using the IBM SPSS; version 21.0. The significance level was set at *P* < 0.05.

## 6. Result

**Figure 1:**
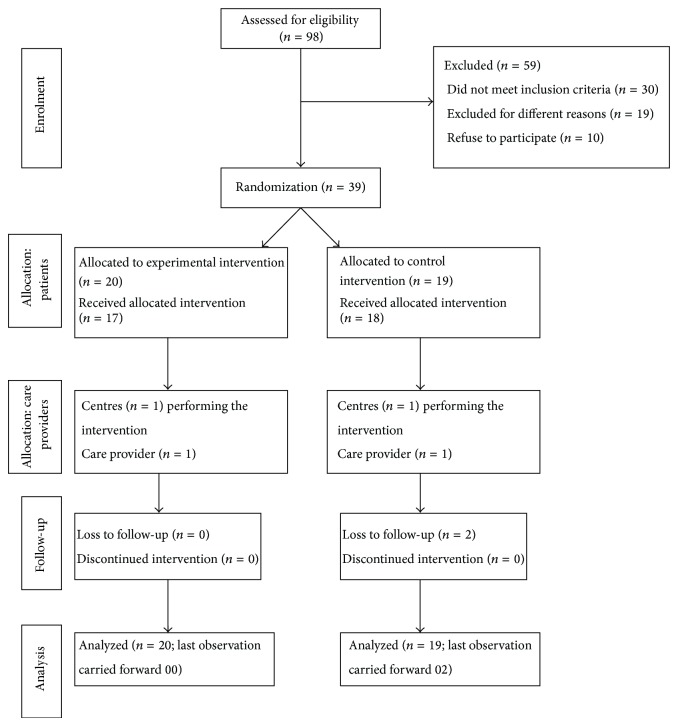
Consort diagram showing the flow of participants during the study.

Ninety-eight poststroke hemiparetic subjects were screened for the eligibility criteria. Fifty-nine subjects (49 did not meet the eligibility criteria and 10 refused to participate) could not be enrolled in the study. Finally, 39 subjects were randomly divided into the experimental group (*n* = 20) and control group (*n* = 19).  Figure 1 shows the consort diagram for the distribution of subjects during the entire study. There were 21 (54%) men and 18 (46%) women with an average age of 42 years. Twenty-two (56%) subjects had ischemic stroke while 17 (44%) had hemorrhage stroke. The mean poststroke duration was 13 months. Further, there were 20 (51%) right paretic and 19 (49%) left paretic study participants. The participants exhibited motor level of 32/66 for upper limb and 20/34 for the lower limb on Fugl-Meyer Assessment. The detailed demographic characteristics and motor status of both the groups are given in Table 1.

Before intervention, there were 6 (30%) in the medium fall risk and the remaining 14 (70%) were in the low fall risk as per the BBS score in the experimental group. In the control group, 7 (37%) subjects were under medium fall risk and the remaining 12 (63%) were at low fall risk. No subject was in the high fall risk (0–20) among both the groups. After intervention, the distribution changed as 100% experimental subjects in the low fall risk. Comparatively, the allocation changed as 5 (26%) control subjects in the medium fall risk and 14 (74%) in the low fall risk. Before the commencement of the study, occasional fall history was reported by one experimental group subject and two control group subjects. However, during the study protocol none of the participants experienced any fall.

**Figure 2:**
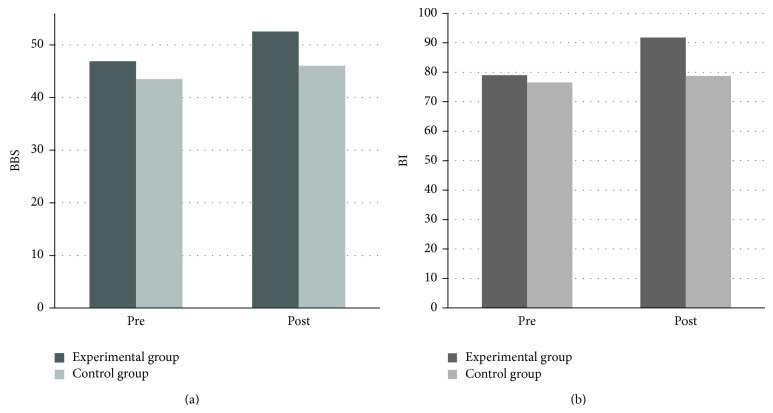
(a) and (b) show changes in the Berg Balance Scale (BBS) and Barthel Index (BI) scores, respectively (mean) between the groups at pre- and postassessment; dark grey bar (experimental group), light grey bar (control group). Significant improvements (increase in BBS and BI scores) were noted for both the measures at postintervention.

After intervention, the BBS and BI score improved significantly (*P* < 0.05) in the experimental group as compared to the control group (Table 2 and Figure 2). However, no significant change was observed for FRT between the groups. Considering the specific items of BBS, both sit-to-stand and stand-to-sit improved 10% in the experimental group compared with 1.5% among the control group subjects. No change was observed for the picking-up objects from the floor in the control group while 9% improvement was noticed in the experimental group. Turning to look behind while standing exhibited 11.50% positive change in the experimental group compared with 1.5% change in the control group. Similarly, placing alternate foot on the floor showed a change of 12.50% in the experimental group in comparison to 1.5% among the control group subjects. Standing unsupported with one foot in front demonstrated an achievement of 20% among the experimental group subjects compared with only 2.75% in the control group. Standing on one leg improved maximum among all the items, it progressed to 27% in the experimental group compared with only 1.5% in the control group.

The improvement of BI items ranged from 10% to 35% in the experimental group compared with 2% to 7% among the control group subjects (feeding, 10% versus 7%; grooming, 35% versus 5%; toilet use, 10% versus 2%; transfer, 10% versus 3%; mobility, 12.5% versus 4%; stairs, 15% versus 2%).

Before intervention, the experimental group exhibited stage 4 both for BRS arm and lower limb which changed to stage 6 and 5-6, respectively, after the intervention. In the control group, prior to the intervention BRS arm was 4 and lower limb was 3-4, and the stages progressed to 4-5 and 3-4, respectively, after intervention. The nonparetic upper limb exhibited median muscle-strength grade range from 3 (3, maximum contraction against gravity; 3+, maximum contraction against gravity with minimal resistance) to 4 (4, maximum contraction against gravity with moderate resistance; 4−, maximum contraction against gravity with moderate resistance incomplete range) (shoulder, 3+; elbow, 4; forearm, 4; wrist, 4). Similarly, the lower limb demonstrated the median muscle-strength grade varied from 3 to 4 (hip, 3+; knee, 4− and ankle, 3).

## 7. Discussion

In poststroke subjects, the usually referred “*sound side*” has impairment of muscle strength, dexterity, and coordination. Unexpectedly, the muscle weakness ranges from grade 3 to 4 [1]. The types of impairment have been explored and the term less-affected side is now preferred. However, the role of motor training for the side has not been investigated yet. The present study was a pilot work that focused on the intervention of the nonparetic side. In the study, motor therapy involving the nonparetic side positively influenced the balance and function. In healthy individuals, successful performance of balance and functional activities needs coordinated participation of both sides of the body. After stroke, subtle motor impairment on the ipsilesional side may affect balance and function. Varied degrees of arm impairments are observed on the ipsilesional side [42]. The grip strength of nonparetic side was found to be one of the risk factors for balance among stroke subjects [43]. However, therapeutic approaches focusing on the nonparetic side for such deficits have never been explored.

Balance and functional tasks are controlled by both sides of the body. Balance and function are related abilities; the good balance provides a base for independent functional performance [44]. After stroke, the imbalance between the affected and nonparetic side induces an asymmetry. Additionally, most of the daily functions are expected to be performed by the nonparetic side. Hence, it is understood that motor impairment of the nonparetic side may contribute to balance and functional deficits [11–13].

Dynamic balance is usually impaired in stroke subjects. Poor muscle strength is one of the additional factor for compromised dynamic balance [45]. Lower extremity weakness is also associated with the dynamic balance [45]. For successful performance of all daily functions a fair amount of dynamic balance is required. In the present study, the postural transition which requires good body strength and stability was improved after the experimental protocol. Further, the improvement in balance might have contributed to greater functional achievement.

Due to various reasons, the stability of affected limb gets impeded. The nonparetic extremity is supposed to take extra efforts to keep the body stable during various postures. The motor training including resistive strengthening may enhance the capability of nonparetic side for stability and balance [7, 46].

All the components of BBS require stability and strength of both sides of the body. In addition to the arms, the lower limbs also play a vital role for maintaining and change in body posture [18]. The strengthening of lower limbs was performed in the present study. This could also be the reason for improvement in balance. After the experimental protocol, some of the BBS components demonstrated positive clinical changes validating the role of motor therapy directed toward the nonparetic side. BBS items such as turning to look behind, placing alternate foot on the floor, standing with one foot in front and standing on one foot all need good strength in lower extremity muscles improved after the experimental protocol. Standing on one foot improved maximally out of all the items.

Every functional activity needs good balance. In stroke, fall frequency increases many folds during key functional activities like mobility and transportation [47]. Maintaining standing position and ambulatory activities were found to have influence on balance and function [48]. Various dynamic postures used in BBS are also the part of day-to-day activities. This explains the positive improvement on BI score in the experimental group in comparison to the controls.

Maintaining balance requires less amount of muscle strength than change of posture. FRT assesses static balance only. After intervention, it demonstrated no significant change.

The nonparetic side is evident to be impaired. Additionally, the affected side gets compensated by the nonparetic side for various functions [49]. Weight bearing asymmetry and dynamic balance could not be improved with external support and authenticates the other neglected factors for poor balance [50]. Only correction of weight bearing asymmetry will not be helpful for poststroke rehabilitation. Further, despite of various researches for balance training, no specific approach is considered to be ideal [51]. Hence, to alleviate the balance dysfunction, the strengthening of the nonparetic side should be included in the management [52].

Varied area of lesion was one of the limitations of this study. Further, no sample size calculation was done for the present study. Stroke-specific functional measure may be used in the future studies. Laboratory measures such as force plate are also recommended to objectively evaluate the balance. Long term studies will be done to understand the achievement of maximum possible muscle strength on the nonparetic side and its impact on balance and function. The study participants reported no adverse event during the course of trial; however, few experimental subjects experienced mild postexercise muscle soreness. Appropriate rest was recommended for those subjects.

## 8. Conclusion 

The motor therapy program comprising strengthening for the nonparetic side and bimanual activities was found to be effective in enhancing balance and function in stroke patients. It is suggested that the nonparetic side should also be incorporated in the motor rehabilitation of chronic stroke patients.

## Figures and Tables

**Table 1:** Demographic characteristics and motor status of the participants.

Characteristics	Experimental group (*n* = 20)	Control group (*n* = 19)	Test statistics
Age in yrs. (Mean ± SD)	44.50 ± 13.59	40.16 ± 14.96	*t*: *P* = 0.349
Gender (male/female)	11/(58%)/8 (42%)	10 (50%)/10 (50%)	*χ* ^2^: *P* = 0.621
Duration of onset (months)	12.72 ± 8.26	13.32 ± 6.68	*t*: *P* = 0.804
Side of involvement (right/left)	9 (45%)/11 (55%)	11 (58%)/8 (42%)	*χ* ^2^: *P* = 0.421
Type of stroke (ischemic/hemorrhagic)	13 (68%)/6 (32%)	9 (45%)/11 (55%)	*χ* ^2^: *P* = 0.140
Area of involvement (frontoparietal/basal ganglia/thalamic/multiple areas/others)	4 (20%)/2 (10%)/2 (10%)/10 (50%)/2 (10%)	6 (31.5%)/4 (21%)/2 (10.5%)/6 (31.5%)/1 (5%)	—
Fugl-Meyer Assessment (upper extremity) (Mean ± SD) *Max. score 66 *	35.65 ± 14.88	29.05 ± 14.85	*t*: *P* = 0.171
Fugl-Meyer Assessment (lower extremity) (Mean ± SD) *Max. score 34 *	21.15 ± 2.99	19.63 ± 3.86	*t*: *P* = 0.177
Use of assistive device/orthosis			
Walking stick	5 (26%)	3 (15%)	*f*: *P* = 0.451
Ankle foot orthosis	9 (45%)	7 (63%)	*χ* ^2^: *P* = 0.605
Shoulder sling	2 (10%)	2 (10.5%)	*f*: *P* = 1

SD: standard deviation.

**Table 2:** Pre- and Postintervention change in outcome measures between the experimental group and control group.

Outcome measure	Pre			Post				
	Experimental Group (*n* = 20)	Control group (*n* = 19)	Test Statistics	Experimental Group (*n* = 20)	Control group (*n* = 19)	95% CI (Difference between the groups postintervention)	*F*	*P* value
BBS (max. score 56) (mean ± SD)	46.90 ± 6.84	43.53 ± 8.67	*t*: *P* = 0.184	52.55 ± 3.07	46.05 ± 8.35	0.631–9.24	5.39	0.026^*^
FRT (in inches) (mean ± SD)	8.02 ± 2.53	7.84 ± 3.13	*t*: *P* = 0.842	9.53 ± 2.14	8.05 ± 3.14	0.932–2.58	0.908	0.347
BI (mean ± SD)	79.00 ± 11.98	76.58 ± 13.75	*t*: *P* = 0.561	91.75 ± 6.74	78.74 ± 15.50	9.79–5.11	4.14	0.049^*^

BBS: Berg Balance Scale, FRT: Functional Reach Test, BI: Barthel Index, SD: standard deviation, CI: confidence interval, *F*: test value for repeated-measures 2-way ANOVA, ^*^significant.
